# Alcohol’s Effects on Breast Cancer in Women

**DOI:** 10.35946/arcr.v40.2.11

**Published:** 2020-06-18

**Authors:** Jo L. Freudenheim

**Affiliations:** 1 School of Public Health and Health Professions, University at Buffalo, Buffalo, New York

**Keywords:** alcohol drinking, breast cancer incidence, breast cancer survival, drinking pattern, women

## Abstract

Globally, more than 2 million new cases of breast cancer are reported annually. The United States alone has more than 496,000 new cases every year. The worldwide prevalence is approximately 6.8 million cases. Although many risk factors for breast cancer are not modifiable, understanding the role of the factors that can be altered is critical. Alcohol consumption is a modifiable factor. Studies of alcohol in relation to breast cancer incidence have included hundreds of thousands of women. Evidence is consistent that intake, even intake of less than 10–15 grams per day, is associated with increased risk of this disease. In addition, evidence, although less extensive, shows that possible early indicators of risk, such as benign breast disease and increased breast density, are associated with alcohol consumption. Evidence is less strong for differences based on geographic region, beverage type, drinking pattern, or breast cancer subtype. Some studies have examined the association between alcohol and recurrence or survival after a breast cancer diagnosis. These findings are less consistent. Public awareness of alcohol as a risk factor for breast cancer is low, and public health measures to increase that awareness are warranted.

## INTRODUCTION

In 1987, the *New England Journal of Medicine* published two reports about alcohol consumption and breast cancer risk.^1^,^2^ In the two reports, both prospective cohorts, alcohol consumption, even at modest levels of intake, was associated with risk of breast cancer. An accompanying editorial indicated that based on the existing epidemiologic studies, approximately 17 at the time, one could conclude “despite variations in study design, population, culture and language of the country of origin, and methods of determining the amount of alcohol ingested, most investigations have found at least a small increase in risk with increases in intake, particularly among premenopausal women.”^3^ Since those landmark papers were published, studies have been conducted among hundreds of thousands of women. Findings of an association between alcohol consumption and an increase in breast cancer risk for women have persisted.

## SCOPE OF THE PROBLEM

Breast cancer affects more than 2 million women each year around the world.^4^ The age-adjusted rate is 46.3 new cases of this disease per year for every 100,000 women. In the United States, more than 496,000 new cases are diagnosed every year, and the age-adjusted incidence is 84.8 per 100,000 women. Globally, 626,679 deaths from breast cancer occur annually, and in the United States, close to 89,000 deaths were reported. The age-adjusted breast cancer mortality rates are 13.0 deaths per 100,000 women globally, and 12.6 deaths per 100,000 women in the United States. It is estimated that the prevalence of breast cancer around the world is 6.8 million cases.

## ALCOHOL AND BREAST CANCER INCIDENCE

A large body of research provides evidence that alcohol is a risk factor for incidence of breast cancer. The World Cancer Research Fund and the American Institute for Cancer Research (WCRF-AICR) collaborated to organize a continuous systematic review of dietary factors in relation to cancer.^5^ The WCRF-AICR reports include examinations of alcohol and breast cancer. In a 2018 update, they concluded that, based on the existing literature (16 prospective studies of premenopausal breast cancer and 34 of postmenopausal disease), alcohol consumption is a “probable cause” and a “convincing cause” for premenopausal and postmenopausal breast cancer, respectively. The meta-analysis showed that for a 10-gram increase in alcohol consumed per day on average, risk increased 5% among premenopausal women and 9% among postmenopausal women. A standard drink contains approximately 14 grams of alcohol.^6^

As noted in the 1987 editorial in the *New England Journal of Medicine*, an association between alcohol and breast cancer was found across geographic locations for a range of beverage types consumed and for a variety of drinking patterns.^3^ Most of the studies on alcohol and breast cancer have been conducted in North America and Europe, but there are some from other locations.

The WCRF-AICR meta-analysis reported some differences by location.^5^ For premenopausal breast cancer, the summary meta-analysis was significant only for North America. Results were similar in magnitude but not statistically significant for analyses of findings from Europe and Asia. For postmenopausal cancer, in the meta-analysis of dose-response, the association was statistically significant only for studies of Europe and North America.

In a study that pooled data from 20 cohorts in the United States, Canada, Europe, Australia, and Japan, no significant heterogeneity was found among studies, although the association between alcohol and breast cancer was stronger for the North American cohorts than for the others.^7^ Even within regions, there can be considerable differences in quantities of alcohol consumption, types of beverages consumed, and intensities of drinking (e.g., frequency of binge drinking, drinking with meals or not). For example, within Europe, drinking patterns vary considerably. In a study of 335,000 women in Europe, of whom 11,600 had invasive breast cancer, a significant, 4% increase in risk was shown for each additional 10 grams of alcohol consumed per day.^8^

Studies of individual European countries, including Italy,^9^ France (among postmenopausal but not premenopausal women),^10^ and the United Kingdom,^11^ but not Greece,^12^ also reported evidence of increased risk. In a case-control study of more than 2,000 cases and 2,000 controls from 3 countries in sub-Saharan Africa, an association between alcohol consumption and risk was reported, despite considerable differences in the prevalence of alcohol consumption in those countries.^13^ In South America, studies in Brazil reported some evidence of an association.^14^,^15^ For studies in Asia, where women’s alcohol consumption generally is lower, results have been inconsistent.^16^–^20^

Few studies have examined the association between alcohol and breast cancer by race/ethnicity. The African American Breast Cancer Epidemiology and Risk (AMBER) Consortium, a pooled analysis of studies of African American women, found a J-shaped association between alcohol consumption and breast cancer risk.^21^ The magnitude of the association for higher intakes of alcohol was similar to results reported in other studies of women of European descent.

Overall, there is strong evidence that alcohol increases breast cancer risk. Evidence is strongest for North America and Europe, where more studies have been conducted, but other regions also show some evidence of a similar association. Much additional research has been done regarding the details of the alcohol consumption (e.g., beverage type, drinking pattern, the participant’s age at the time of consumption) and the details of the breast cancer (e.g., tumor subtype). These findings are less consistent.

Variability in findings may be a function of the small sample size of some studies, for instance, in those studies that examined associations between alcohol consumption for breast cancer by subtype (e.g., estrogen receptor–positive or –negative). In addition, alcohol consumption can be difficult to assess for a variety of reasons, including difficulty recalling usual intake, change in consumption over the lifetime, and response bias. In this context, the consistency of the findings regarding overall risk of breast cancer associated with alcohol consumption is noteworthy.

### Beverage Type

Several studies of alcohol and risk examined whether there are differences depending on the beverage consumed: wine, beer, or spirits. The pooled analysis of 20 cohorts reported no difference in risk based on the beverage type.^7^ The Million Women Study in the United Kingdom reported similar associations for those who drank wine only and for those who consumed other drinks.^11^ In the WCRF-AICR meta-analysis, only beer was associated with a statistically significant increase in risk among premenopausal women, and only wine was associated with risk among postmenopausal women.^5^ However, in all of the studies, there was an indication of increased risk with each of the beverages, even if not statistically significant. In addition, the evidence was that there was not a statistical difference of the association with each of the three types of beverage for both premenopausal and postmenopausal analyses. Some studies provided evidence of a stronger effect for a particular beverage, but most of the evidence pointed to effects from any alcoholic beverage.

### Drinking Pattern

When examining the effects of alcohol consumption on health and disease, how participants consumed the alcohol must be considered. Not only the absolute quantity consumed, but also the intensity of consumption may have biological effects. For example, the effects of an average consumption of seven drinks per week may differ for consumption of one drink daily and for seven drinks on one day once per week.

Just a few studies have examined drinking intensity. In the Nurses’ Health Study I (NHS), binge drinking (defined as six or more drinks in one day) was associated with increased risk, even after adjusting for total consumption.^22^ The frequency of alcohol consumption was not associated with risk in that cohort after adjusting for total consumption. In the Sister Study, a cohort of women with a family history of breast cancer, self-report of ever binge drinking (defined as four or more drinks in one sitting) or ever having blacked out while drinking were associated with increased breast cancer risk.^23^ These associations were not adjusted for overall alcohol intake.

Even among people who drink lightly, evidence of increased risk has been reported. In a systematic review of light drinking, which used the World Health Organization definition of less than 21 grams of alcohol consumed per day, Shield and colleagues found consistent evidence of increased risk.^24^ In a meta-analysis, Choi and colleagues found statistically significant increases in risk of 4%, 9%, and 13% for individuals who drank less than 0.5 drinks per day, less than or equal to 1 drink per day, and 1 to 2 drinks per day, respectively; in this analysis, one drink was defined as 12.5 grams of alcohol.^25^ There is no evidence of a lower threshold for an effect of alcohol consumption on risk of breast cancer. Collectively, results from these studies on intake indicate that drinking pattern may affect risk, as drinks per drinking day are associated with increased risk even after adjusting for total intake.

### Breast Cancer Subtype

Breast cancer can be classified into subtypes by tumor markers. The subtypes may have different risk factors, and they are different in terms of aggressiveness, treatment, and prognosis. A number of studies have examined the association between alcohol consumption and invasive breast cancer by subtype.

In the European Prospective Investigation into Cancer and Nutrition (EPIC) study, which examined a cohort of more than 360,000 women from 23 centers in 10 countries in Europe, the association between alcohol consumption and risk was stronger for women with estrogen receptor–positive tumors than for those with estrogen receptor–negative tumors.^26^ In a report on postmenopausal breast cancer from the Million Women Study in the United Kingdom, no heterogeneity by estrogen receptor status was found for the association between alcohol consumption and risk.^27^ A pooled analysis of 20 cohort studies, which comprised more than 1 million women, reported no difference in the associations of alcohol and estrogen receptor–positive tumors or of alcohol and estrogen receptor–negative tumors.^7^ Finally, in the systematic review by the WCRF-AICR, the findings for postmenopausal cancer indicated an increase in risk for estrogen receptor–positive tumors but not for estrogen receptor–negative tumors.^5^

In one study, alcohol consumption and risk of human epidermal growth factor receptor 2 (HER2)–positive and triple-negative breast cancers were compared to risk of estrogen receptor–positive tumors.^28^ Alcohol consumption was associated with a lower risk of HER2-positive tumors and no difference in the risk of triple-negative tumors, as compared to its association with risk for estrogen receptor–positive tumors. In an analysis of data from the AMBER Consortium of African American women, the association between alcohol consumption and risk was stronger for estrogen receptor–negative, progesterone receptor–negative, and HER2-negative tumors than for tumors with positive receptor status.^21^ Overall, findings from studies of associations between alcohol consumption and breast cancer subtypes have been inconsistent.

### Period of Exposure

Alcohol consumption patterns generally vary during the life span, and effects of exposures may differ depending on the stage of breast development when the drinking occurred. A number of studies have examined risk associated with alcohol consumption at particular time periods, especially during adolescence and early adulthood.

The NHS II, a prospective study of women ages 24 to 44 at baseline, reported an 11% increase in breast cancer risk associated with consumption of 10 grams of alcohol per day between menarche and first pregnancy, adjusting for subsequent intake.^29^ A similar increase in risk was observed for consumption of alcohol after the first pregnancy, adjusting for intake before that time. In NHS I, a cohort of women ages 30 to 55 at baseline, there was an 8% increase in risk associated with 10 grams of alcohol consumed per day between ages 18 and 40, even after adjusting for consumption after age 40.^22^ For consumption after age 40, there was a 7% increase in risk, after adjusting for earlier intake.

Benign breast disease is associated with increased breast cancer risk and may be an early indicator of risk. In the NHS II, evidence indicated a 15% increase in risk of benign breast disease for each additional 10 grams per day of alcohol consumed during adolescence.^30^ Another study of young women reported a 50% increase in risk of benign breast disease for each additional drink per day during the period of ages 9 to 15.^31^ In one study, associations for alcohol with risk were similar for pre-cancerous conditions as for invasive breast cancer.^32^

The EPIC cohort study examined the association between risk and alcohol consumption for parous women before their first, full-term pregnancy compared with women who did not begin drinking until after their first pregnancy.^8^ Point estimates were similar but there was a significant association only for those who started drinking before their first pregnancy. In addition to intake during adolescence and young adulthood, even exposure to alcohol *in utero* may predispose to increased risk. Evidence from animal models indicates that ethanol exposure *in utero* can lead to increased breast tumorigenesis in the adult offspring when exposed to carcinogens.^33^

These studies indicate that the association of lifetime alcohol consumption with breast cancer risk may be different depending on when the alcohol was consumed. Evidence shows, with some inconsistency among studies, that consumption in adolescence and before a first pregnancy may particularly affect risk.

### Breast Density

Breast density is a measure of breast tissue from radiography. It is associated with subsequent breast cancer and is one of the strongest breast cancer risk factors.^34^,^35^ Understanding factors related to increased density may provide insight into early stages of carcinogenesis. A number of cross-sectional analyses have shown that alcohol consumption is associated with increased breast density. In a study in Germany, consumption of more than 10 grams of alcohol per day was associated with increased risk of high mammographic density.^36^ Similarly, increases in risk of increased breast density were associated with alcohol drinking in Japan,^37^ Sweden,^38^ and the United States in Hawaii^39^ and New York City.^40^ There was a nonsignificant association in a study in China.^41^

In some studies, the association between alcohol consumption and risk varied depending on other breast cancer risk factors. In the Swedish study, the association was strongest for the group that also had other factors that predicted increased risk of breast cancer.^38^ In a multicultural population in New York City, the association was strongest among individuals who had lower body mass index.^40^ In a study of Mexican women, alcohol use was associated with increased breast density.^42^ In a study of NHS II participants, no association was found between breast density and alcohol consumption.^43^ A meta-analysis of studies reported an association between increased breast density and higher levels of alcohol consumption.^35^ Although these reported findings are not consistent, effects of alcohol consumption on breast density may be one mechanism for the associations with risk for breast cancer.

### Diet

A number of studies have examined alcohol consumption in concert with other known breast cancer risk factors. In particular, there has been study of interactions of alcohol with other dietary factors such as folate and other B vitamins, which play a role in alcohol metabolism. Alcohol negatively affects folate status, impacting folate absorption and metabolism and increasing folate excretion.^44^ A systematic review reported evidence of interaction between alcohol and folate in relation to breast cancer risk.^45^ Breast cancer risk decreased with increased folate consumption among individuals who drank heavily but not lighter drinkers.

Several recent studies examined plasma folate as a measure of vitamin status. In the NHS II, there was an interaction between alcohol and plasma vitamin concentrations, with a trend toward plasma folate being protective for breast cancer risk among individuals who consumed greater amounts, but not among those consuming lesser amounts of alcohol.^46^ However, in the NHS I, plasma folate was not associated with breast cancer risk and did not vary by alcohol consumption.^47^

Further, in the EPIC cohort study in Europe, no interaction was found for alcohol and plasma folate consumption in relation to breast cancer risk.^48^ This study found some evidence of an interaction of alcohol and plasma vitamin B_12_ consumption in relation to breast cancer risk; vitamin B_12_ also is a cofactor in one-carbon metabolism. A study that examined the Women’s Health Study cohort found no interaction between plasma concentrations of B vitamins and alcohol consumption in relation to risk.^49^ A systematic review found evidence for an association between higher levels of folate consumption and decreased risk of breast cancer among participants with moderate or high alcohol intake.^50^ Collectively, these results show that diet, particularly vitamins related to one-carbon metabolism, may modify the association between alcohol and the risk for breast cancer.

### Genetic Factors

Several studies have examined genetic variation in the association between alcohol consumption and breast cancer risk. There have been several studies of the genes that code for the alcohol dehydrogenases (ADH), which are critical enzymes for alcohol metabolism. In a cohort in the Netherlands, variants in the genes for ADH were not associated with breast cancer risk nor did they modify the risk associated with alcohol consumption.^51^ The NHS I reported similar findings; the association between alcohol consumption and risk for breast cancer was not modified by genetic variation in ADH.^52^ There was, however, evidence that an association between alcohol and steroid hormone levels differed depending on ADH genotype.

A Danish cohort study examined variation in the *CYP19A1* gene, which codes for aromatase, an enzyme important to estrogen metabolism.^53^ Although these researchers found an interaction of genetic variation with blood steroid hormones with acute alcohol consumption, they found no evidence of an association of the genetic variant with breast cancer risk. Among women who have the *BRCA1* or *BRCA2* genes, mutations that confer a particularly elevated risk of breast cancer, alcohol was not associated with breast cancer risk.^54^ Overall, the evidence for genetic factors modifying the association between alcohol consumption and the risk for breast cancer is not strong.

### Other Potential Modifying Factors

Understanding of whether other factors modify the observed association between alcohol consumption and breast cancer is another area of active research. In a pooled analysis, alcohol was positively associated with risk among both nulliparous and parous women.^55^ Point estimates of risk were similar and not significantly different for the two groups. There is some evidence of a stronger association between alcohol and breast cancer risk among women receiving hormone therapy as compared to those not receiving hormone therapy, particularly the risk for estrogen receptor–positive breast cancer.^56^ Further examination of modifying factors such as other dietary factors, body mass index, level of physical activity, and smoking is warranted.

## ALCOHOL AND SURVIVAL AFTER DIAGNOSIS

Although most of the research regarding the association between consuming alcohol and the risk for breast cancer has focused on incidence, some studies have examined the effects of alcohol on survival after a breast cancer diagnosis. Studies used different time frames (before or after diagnosis) for the alcohol consumption and different outcome measures, such as breast cancer recurrence, breast cancer–specific survival, and all-cause mortality. Most studies did not distinguish by breast cancer subtype, which can affect prognosis.

A meta-analysis of 11 studies found evidence of improved survival after breast cancer diagnosis among individuals who reported any prediagnostic alcohol consumption, when compared with those who reported none.^57^ The association differed somewhat by the estrogen receptor status of the tumor, with some evidence of reduced all-cause mortality for women with estrogen receptor–negative disease and no association with mortality in those with estrogen receptor–positive disease. Studies of lifetime alcohol intake found no association with all-cause mortality or death from breast cancer (breast cancer–specific mortality).^58^,^59^

In the National Institutes of Health (NIH)-AARP Diet and Health Study cohort, alcohol consumption at the study baseline was not statistically significantly associated with breast cancer–specific survival.^60^ In the Women’s Health Initiative, there was no association between prediagnostic alcohol consumption and breast cancer–specific or all-cause mortality.^61^ There was some evidence of decreased breast cancer–specific mortality for estrogen receptor–negative tumors. Among breast cancer patients from the Moffitt Cancer Center, self-reported alcohol consumption one year before diagnosis was associated with improved breast cancer–free survival.^62^ Another study of women in the United States reported that prediagnostic alcohol intake was associated with an increased risk of breast cancer–specific mortality.^63^

Alcohol consumption pattern may affect mortality as well as incidence. In a study in western New York among women who had postmenopausal breast cancer, drinking intensity before diagnosis was associated with prognosis.^59^ Participants who drank four or more drinks per drinking occasion had increased mortality from breast cancer and from all causes, and participants who drank fewer drinks per drinking occasion had decreased mortality from both breast cancer and all causes.

Few studies have examined alcohol consumption following a breast cancer diagnosis. One study reported an increased risk of breast cancer recurrence with alcohol consumption after diagnosis among premenopausal but not postmenopausal women.^64^ In another study, investigators found no association between postdiagnostic intake and breast cancer–specific mortality.^63^ There was better overall survival for those with greater postdiagnostic alcohol consumption. Findings regarding alcohol consumption and prognosis after a breast cancer diagnosis are not consistent. More research is needed to examine alcohol consumption, including patterns of consumption, following diagnosis.

More analyses regarding breast cancer subtype and treatment are required to better understand a possible role of alcohol consumption following diagnosis. Recent studies examining alcohol consumption and the efficacy of breast cancer treatments have not found any effect of alcohol consumption on radiotherapy^65^ or on adjuvant hormone therapy.^62^ More data regarding in-depth analysis of alcohol consumption both before and after diagnosis are needed, along with more research examining the total amount of alcohol consumed, drinking patterns in relation to outcomes, and the effects of drinking alcohol during treatment.

## MECHANISMS FOR ALCOHOL EFFECTS

The role of alcohol consumption in breast carcinogenesis is a complex process likely acting through a number of mechanisms. Although alcoholic beverages contain a variety of compounds, for breast carcinogenesis, alcohol itself appears to be the more important carcinogen,^66^ consistent with the finding that overall, risk does not differ based on the type of beverage consumed. However, much is not understood regarding the underlying mechanisms for alcohol and breast carcinogenesis. Potential mechanisms include oxidative stress, cell proliferation, effects on hormones, particularly steroid hormones, and effects on one-carbon metabolism.

Alcohol likely contributes to carcinogenesis partly through oxidation from alcohol metabolism and through oxidative stress from production of the alpha-hydroxyethyl radical, a reactive oxygen species.^67^ Alcohol is metabolized to acetaldehyde, classified as a carcinogen by the International Agency for Research on Cancer (IARC), part of the World Health Organization, in 2010.^67^ Although production of acetaldehyde from alcohol primarily occurs in the liver, it also occurs in breast tissues.

There is in vivo evidence that acetaldehyde can concentrate in mammary cells following a single exposure. In an animal model, acetaldehyde accumulated and persisted in higher concentrations in breast tissue than in blood.^68^ Adverse effects of acetaldehyde include DNA adduct formation, oxidation, and altered DNA methylation.^67^ Further, in vitro, at low concentrations, alcohol can increase cell proliferation, including proliferation of breast cells.^69^ Higher concentrations of alcohol and red wine exposure may reduce cell proliferation.

In addition to the carcinogenic effects of alcohol consumption and acetaldehyde on breast tissue, alcohol consumption’s effects on hormones also may contribute to cancer in the breast. There are both acute and chronic effects of alcohol on steroid hormone level. At doses of even 15 to 30 grams of alcohol per day, serum estrogens increase.^24^ In one study of premenopausal women, alcohol consumption was associated with plasma estrogens, but not androgens, when measured during the luteal phase. Neither hormone was associated with alcohol during the follicular phase.^70^ In that same cohort, urinary estradiol measured at the mid-luteal phase was more than 20% higher in women who drank more than 15 grams per day, when compared with those who did not drink.^71^ Further, a mediation analysis provided evidence that changes in hormones associated with alcohol consumption may explain part of the relationship between alcohol and breast cancer.^72^

Altered DNA methylation also contributes to carcinogenesis. Alcohol significantly affects one-carbon metabolism, including DNA methylation, in part by effects on folate status, as discussed previously. Studies that examined DNA methylation in breast tumors made comparisons based on drinking history and found differences by the amount of alcohol consumption.^73^,^74^ Another study found some evidence of these differences in normal, noncancerous breast tissues.^75^ Alcohol’s effects on estrogen also may play a role in altered DNA methylation. There is evidence that higher concentrations of the steroid hormone affect DNA methylation.^24^

Other possible mechanisms for an effect of alcohol on carcinogenesis in general and breast cancer in particular are still emerging. For example, the microbiome in the mouth and gut may affect breast cancer risk,^76^,^77^ and alcohol can affect the microbiome.^78^,^79^ Alcohol likely has other effects on breast carcinogenesis, including effects on metastasis, angiogenesis, and cancer stem cells, affecting both cancer initiation and tumor aggressiveness.^80^

Alcohol’s effects on oxidative stress, cell proliferation, steroid hormones, and one-carbon metabolism may explain, in part, the observed associations with breast cancer risk. Additional research is needed regarding these and other mechanisms, including research on those specific to tumor subtypes and mechanisms for exposures following a breast cancer diagnosis.

## PUBLIC AWARENESS OF RISK

A limited number of studies have examined public understanding of alcohol and breast cancer. In a study of women attending a breast screening clinic in the United Kingdom, only 19% were aware that alcohol consumption is a breast cancer risk factor.^81^ Among university students in a survey conducted in 23 countries around the world, overall, 3.3% were aware of alcohol consumption as a breast cancer risk factor.^82^ Although awareness was highest in the United States, only 10% of students correctly identified alcohol consumption as a risk factor.

Awareness tends to be greater among women who have been diagnosed with breast cancer, with resulting lower alcohol intake in that group. In a systematic review, 62% to 97% of participants adhered to recommendations to limit alcohol consumption in a study of women completing initial treatment for breast cancer.^83^ These studies were conducted primarily in the United States; a small number of participants were in Europe. In spite of the strength of the overall evidence connecting alcohol consumption to breast cancer,^5^,^67^ there is little public awareness of alcohol consumption as a breast cancer risk factor.

## RECOMMENDATIONS

Reduction of alcohol consumption could measurably affect the burden of disease related to breast cancer. Based on global data of the prevalence of alcohol consumption and of the incidence rate of breast cancer, an estimated 144,000 new cases of breast cancer and 38,000 breast cancer deaths annually are accounted for by alcohol consumption, which is 8.6% of all incidence and 7.3% of mortality.^24^ The magnitude of effect of a decrease in consumption in a particular region depends on the prevalence of alcohol consumption in that region. For example, in Australia, it has been estimated that any regular consumption of alcohol accounts for 12.6% and 6.6% of premenopausal and postmenopausal breast cancer, respectively.^84^ Alcohol consumption accounts for 12% of breast cancer in the United Kingdom.^11^ In the United Kingdom, regular consumption of each additional drink per day accounts for 11 additional breast cancers per 1,000 women in their lifetime, up to age 75.^11^ As further indication of the effect, one estimate is that the increase in cancer risk for drinking one bottle of wine per week is approximately equivalent to smoking 10 cigarettes per week, with breast cancer accounting for most of that increase.^85^

Although the evidence is strong for an increase in breast cancer with alcohol consumption, some areas of research still require further attention. A better understanding of the roles of drinking pattern, or drinking intensity, in relation to total consumption is needed. More studies of alcohol consumption and breast cancer subtypes would help increase insight into the relationship. A clearer understanding of the effects of exposures in early life, including *in utero* exposure, is warranted. Examination of how other breast cancer risk factors (e.g., physical activity, body mass index, smoking, reproductive history) interact with alcohol consumption in relation to both breast cancer risk and prognosis is needed. More studies of the association by race/ethnicity, by age at diagnosis, and conducted in regions outside of Europe and North America would contribute to our understanding. Additional research linking epidemiological information with biological information regarding the role of alcohol in carcinogenesis could enhance the ability to leverage this important relationship toward prevention efforts.^44^ Further, additional study is needed of the effects of alcohol consumption, both before and after diagnosis, on breast cancer recurrence, breast cancer–specific mortality, and overall mortality.

Given the strength of the evidence linking alcohol to breast cancer, increasing awareness of risk is critical. It is time for a clear public health message identifying the role of alcohol in breast carcinogenesis and indicating that that there is no apparent lower threshold of effect. Consumption levels of less than one drink per day are associated with increased risk. Further, drinking alcohol affects risk at all phases of life, including early and late life. The science is consistent and clear, but awareness is low. It is time for a focus on developing public understanding of alcohol, which is a very common exposure, and its connection with increased risk of breast cancer.
